# Biofortification of oil quality, yield, and nutrient uptake in Indian mustard (*Brassica juncea* L.) by foliar application of boron and nitrogen

**DOI:** 10.3389/fpls.2022.976391

**Published:** 2022-08-23

**Authors:** Salwinder Singh Dhaliwal, Vivek Sharma, Arvind Kumar Shukla, Manmeet Kaur, Vibha Verma, Prabhjodh Singh Sandhu, Amnah Mohammed Alsuhaibani, Ahmed Gaber, Akbar Hossain

**Affiliations:** ^1^Department of Soil Science, Punjab Agricultural University, Ludhiana, India; ^2^Indian Council of Agricultural Research (ICAR) Indian Institute of Soil Science, Bhopal, India; ^3^Department of Plant Breeding and Genetics, Punjab Agricultural University, Ludhiana, India; ^4^Department of Physical Sport Science, College of Education, Princess Nourah bint Abdulrahman University, Riyadh, Saudi Arabia; ^5^Department of Biology, College of Science, Taif University, Taif, Saudi Arabia; ^6^Department of Agronomy, Bangladesh Wheat and Maize Research Institute, Dinajpur, Bangladesh

**Keywords:** mustard food quality, Indian mustard, boron, sulfur, nitrogen, yield, nutritional uptake

## Abstract

Indian mustard (*Brassica juncea* L.) is an essential oilseed crop that offers important nutrients to human beings. However, the concurrent micronutrient deficiencies including boron (B), sulfur (S), and nitrogen (N) could pose a significant threat to public health. Therefore, this study was conducted at the Punjab Agricultural University, Ludhiana, with nine treatments, i.e., T_1_-Control (recommended NPK only), T_2_- borax (0.5%) at flowering, T_3_-borax (1.0%) at flowering,T_4_- borax (0.5%) + urea (1.0%) at flowering,T_5_-borax (1.0%) + urea (1.0%) at flowering, T_6_-borax (0.5%) at flowering + capsule formation, T_7_-borax (1.0%) at flowering + capsule formation, T_8_-borax (0.5%) + urea (1.0%) at flowering + capsule formation, T_9_-borax (1.0%) + urea (1.0%) at flowering + Capsule formation, replicated three times in a randomized block design for 2 years (2020–2021 and 2021–2022). The foliar application of borax (1.0%) + urea (1.0%) at the flowering and capsule formation stage (treatment T_9_) was highly efficient in increasing food quality parameters such as crude fiber, total soluble solids (TSS), and protein content with maximum values of 3.77, 24.9, and 27.53%, respectively. Also, maximum yields of seed as well as stover for treatment T_9_ were 1.376 and 6.625 kg ha^−1^, respectively. Similarly, the results for B, S, and N concentrations in seed (27.71 mg kg^−1^, 17.69 mg kg^−1^, and 2.35%), as well as stover (25.92 mg kg^−1^, 17.31 mg kg^−1^, and 0.33%), were maximum in treatment T_9_. Also, B, S, and N uptake by seed (38.18 g ha^−1^, 24.40 g ha^−1^, and 32.05 Kg ha^−1^) and stover (172.55 g ha^−1^, 115.44 g ha^−1^, and 21.99 Kg ha^−1^) were maximum for the treatment T_9_ involving borax (1.0%) + urea (1.0%) at the flowering and capsule formation stage. Whereas, the concentration and uptake decreased in the treatments involving the sole application of borax and urea. Therefore, the application of borax (1.0%) and urea (1.0%) at the flowering and capsule formation stage significantly improved the quality parameters, seed and stover yield, nutrient concentration, and uptake over control and could be used to alleviate the B, S, and N deficiency in Indian mustard.

## Introduction

Indian mustard (*Brassica juncea* L.) belonging to the family Brassicaceae is one of the important oilseed crops and is currently ranked as the world's third important oil seed crop in terms of production and area. Rapeseed and mustard are the main oilseed crops grown in the rabi season in India (Kumar et al., 2014). Rabi season crops also known as winter crops are agricultural crops that are sown in winter and harvested in the spring in India. They are sown around mid-November, preferably after the monsoon rains are over, and harvesting begins in April or May. Oil content in rapeseed and mustard varies from 33 to 46% and average oil recovery is around 32 to 38%. The production of oil along with good quality forage such as stems and leaves, owing to their low fiber and high protein concentration, increases the importance of Indian mustard (Bañuelos et al., 2002). India is the third largest producer of rapeseed mustard with 68.2 lakh tons of production; however, the average yield of rapeseed mustard in India is only 1,089 kg ha^−1^. Also, rapeseed-mustard stands next to groundnut in the oilseed economy, contributing to 20% of the total oilseed production in India. Indian mustard is known as Raya and is considered a vital oil-producing crop among Brassica in India (Meena et al., 2018). Rajasthan, Uttar Pradesh, Haryana, Madhya Pradesh, and Gujarat cover more than 80% of acreage under mustard (Kumar et al., 2009). Oil quantity and quality are the most important parameters of Indian mustard, being largely controlled by the mineral fertilization of the plants (Chandan et al., 2019).

Indian mustard is highly sensitive to boron (B), nitrogen (N), and sulfur (S) deficiency, thereby resulting in a decreased growth, yield, and productivity of the crop (Vanisha et al., 2013; Sanwal et al., 2016). The introduction of high-yielding cultivars increases cropping intensity, whereas the application of micronutrient-free fertilizers and limited the addition of organic manures leads to B deficiency in most of the Indian soils. Boron deficiency is one of the major constraints to crop production (Sillanpaa, 1982; Dhaliwal et al., 2022). Its deficiency has been realized as the second most important micronutrient constraint in crops on the global scale (Ahmad et al., 2012). Boron has emerged as an important micronutrient in Indian agriculture in the context of the spread of its deficiency (Sathya et al., 2009; Arora and Chahal, 2015). About one-third of the cultivated soils in India are deficient in B (Gupta et al., 2008). In India, B deficiency was initially reported at 2% in the year 1980 (Katyal and Vlek, 1985), which has now increased to 52% (Singh, 2012; Singh and Goswami, 2014).

The presence of B is mainly associated with meristematic activity, auxin, cell wall, and protein and pectin metabolism, maintaining correct water relations within the plant, sugar translocation, fruiting processes, and so on (Kandil et al., 2020). Boron is also closely related to the functions that calcium performs in the plant. It has also been suggested that B is necessary for the lignin polymerization process. Apart from major plant nutrients, B plays an important role in the production phenology of mustard, and this crop responds to applied B as reported by Yadav et al. (2016). On the contrary, the absence of B causes stunting and deformation of the growing tips, which can lead to tip mortality, brittle foliage, and yellowing of lower leaf tips. In humans and animals, B deficiency causes the immunological function to deteriorate, as well as increased mortality risk, cell damage, and toxicity. Additionally, S deficiency is increasing day by day with the intensification of agriculture. The fertilizer-responsive varieties have accelerated the depletion of S reserves from the soil. The continuous use of major plant nutrients, such as NPK through chemical fertilizers has resulted in the depletion of soils of their secondary and micronutrient reserves which directly affects the growth of crops. The visual symptoms of S deficiency in oilseed crops are very specific and can be treated in the field throughout the growing season. During flowering, the characteristic changes observed during S deficiency are the color and shape of the petals (Chahal et al., 2018). Severe deficiency of S leads to reduced growth, which is particularly associated with a reduced epidemic rate (Kumar et al., 2011). Crops deficient in S are generally small and spindly with short and slender stalks, delayed cereal maturity, poor nodulation in legumes, reduced nitrogen fixation, and reduced maturity (Kumar et al., 2018). On the contrary, S deficiency in humans can lead to obesity, heart disease, Alzheimer's, and chronic fatigue.

Thus, micronutrient deficiencies in Indian soils have deteriorated the yield and nutrient content of various oilseed crops, making them unfit for human consumption (Senthilkumar, 2018). Moreover, a systematic review has been compiled regarding the shortage of micronutrient concentrations affecting the yield of different agricultural crops (Tripathi et al., 2015). In addition, N is a major nutrient that holds a crucial role in cell division, growth, photosynthetic activity and protein synthesis, which acts as a base in the improvement of yield, and accumulation of nutrients, as well as the quality of oilseed crops, including Indian mustard (Yasari and Patwardhan, 2006; Gheith et al., 2022). Similarly, the translocation process of elements acts as a dominant factor controlling their distribution in different parts of crops (Olama et al., 2014; Abdelsalam et al., 2019). The translocation and accumulation of nutrients vary due to variation in mobility and competition of metals with other nutrients within the plant system (Tibbett et al., 2021). Thus, a balanced amount of nutrient application is required for optimum yield and nutrient concentration of crops.

The mustard crop is very much responsive to B (Mengel and Kirkby, 1987) and N applications (Kumar et al., 2017). Till now, various methods including soil, foliar, and seed treatment of B and N application to crops have been reported to alleviate their deficiency (Jha and Warkentin, 2020). Foliar application is the greatest strategy to increase the micronutrient content in crops among different biofortification techniques because nutrients are supplied to the leaves at the appropriate growth stages (Poblaciones and Rengel, 2017; Doolette et al., 2018; Kandil et al., 2022). The rapid absorption promotes nutrient translocation in the edible seed part while also preventing nutrient loss in the environment. Furthermore, it promotes plant growth even in less favorable weather conditions. Recent research showed that a small amount of nutrients, particularly B and N supplied through the foliar spray, has resulted in a significant increase in the crop yield (Choudhary and Bhogal, 2017; Zalak et al., 2020). Additionally, it is well-known that oilseed crops require high N and an optimum level of B for the production of a sustainable yield. Thus, the supply of B and N along with fertilizers in adequate amounts is essential for the higher yield and quality of oilseed crops. Thus, the current experiment aimed to determine the most efficient and economical B and N treatments to enhance the oil quality, yield, and concentration, as well as uptake of nutrients, in Indian mustard.

## Materials and methods

### Site specification and characteristics

The present study was done during two consecutive Rabi seasons of 2020–2021 and 2021–2022 at the experimental farm, Department of Soil Science, PAU, Ludhiana, Punjab (30° 56′ N, 75° 52′ E, and 247 m above mean sea level) in Indo-Gangetic plains of north-western India during the months of November to April. The pH and EC of the soil were calculated following the method described by Jackson (1973). The wet combustion method was used to determine the organic carbon (OC) content in the soil (Dhaliwal et al., 2021d). The experimental sandy loam soil possessed a pH of 7.30, an EC of 0.35 dS m^−1^, and an OC of 0.38%. Initial micronutrient (Zn, Cu, Fe, and Mn) levels in the soil were determined using the instrument AAS (Varian AAS-FS 240 model) (Lindsay and Norvell, 1978) where the values were 1.20, 0.88, 5.32, and 4.17 mg kg^−1^ for Zn, Cu, Fe, and Mn, respectively. The initial levels of hot water soluble B determined by the azomethalin-H colorimetric method (Wolf, 1971) and available S by the turbidimetric method (Chesnin and Yien, 1950) were found to be 0.45 and 9.15 mg kg^−1^, respectively. The alkaline KMnO_4_ method, the Olsen extractable P method, and the neutral ammonium acetate method were used to estimate the available N, P, and K, respectively (Merwin and Peech, 1950; Jackson, 1973), where the values were 0.084%, 26.52 Kg ha^−1^, and 7.95 Kg ha^−1^ for N, P and K, respectively. The region exhibited a subtropical climate along with hot, rainy summers as well as dry winters. The annual rainfall ranges from 400 to 600 mm, where the months of July to September receive the maximum rainfall of around 70% (Dhaliwal et al., 2021b).

### Treatment details

The study was performed with nine treatments, i.e., T_1_-Control (recommended NPK only), T_2_- borax (0.5%) at flowering, T_3_-borax (1.0%) at flowering,T_4_- borax (0.5%) + urea (1.0%) at flowering,T_5_-borax (1.0%) + urea (1.0%) at flowering, T_6_-borax (0.5%) at flowering + capsule formation, T_7_-borax (1.0%) at flowering + capsule formation, T_8_-borax (0.5%) + urea (1.0%) at flowering + capsule formation, T_9_-borax (1.0%) + urea (1.0%) at flowering + capsule formation, replicated three times in randomized block design for 2 years (2020–2021 and 2021–2022). During planting, the recommended dose of 100 Kg of N and 30 Kg of P_2_O_5_ per hectare were applied as a base through urea and single superphosphate, respectively. The sowing of mustard variety RLC 3 was done in November using the drill method with the inter-row distance of 10 to 15 cm, an intra-row spacing of 30 cm, and a plot size of 4.0 m × 4.8 m = 19.2 m^2^. On the 127th day of physiological maturity, they were manually harvested in April where the seeds, as well as stover samples, were collected for examination.

### Growth parameters

At physiological maturity, observations about growth parameters, such as plant height and the number of capsules per plant, were recorded by averaging the values of the five plants in each plot. The height of the plant was measured with the help of a meter scale from the base to the tip of the plant. Average plant height was computed and expressed in cm. The total number of capsules was also counted manually.

### Proximate composition analysis of Indian mustard seeds

This involved the determination of the percentage of moisture, total ash, crude fiber, total soluble solids (TSS), and protein content in Indian mustard. Proximate composition was determined by the AOAC (1995) standard method.

#### Percentage of moisture

The AOAC (1995) technique was used to determine the moisture content. About 2 g of the sample was weighed in an aluminum dish that had been cleaned and dried properly. The sample was dried at 130 ± 3°C for 1 h in an oven until it reached a consistent weight and then cooled to room temperature. The weight loss after cooling the sample was calculated as the percentage of moisture content by using Equation (1).


(1) $$\begin{array}{r} Moisture\;\left(\%\right)=\frac{M_{2}-M_{3}}{M_{2}-M_{1}}\times 100 \end{array}$$

where M_1_ = mass of empty dish; M_2_ = mass of dish and sample (before drying); and M_3_ = mass of dish and sample (after drying).

#### Total ash

The amount of ash in the sample was assessed through the AOAC (1995) technique where a 5-g sample in a porcelain dish was burned on a hot plate until blackened. It was then cooled and kept in a muffle furnace at 550°C to produce light gray-colored ash. It was then cooled and weighed. The residual weight was recorded, and the percent ash was computed as follows (Equation 2):


(2) $$\begin{array}{r} Ash\;\left(\%\right)=\frac{M_{2-M_{1}}}{M}\times 100 \end{array}$$

where M_1_ = mass of the dish before burning; M_2_ = mass of dish and sample after burning, and M = sample mass.

#### Crude fiber

The method outlined in AOAC (1995) was used to determine crude fiber. About 2 g defatted sample was placed in a beaker containing 200 ml of 1.25% H_2_SO_4_ and heated for 30 min. After 30 min, the solution was filtered using Whatman filter paper no. 54 and the residue was rinsed with hot distilled water. The residue was then heated for 30 min in a 1.25% sodium hydroxide solution. The contents were again filtered through Whatman filter paper no. 54 and rinsed with hot distilled water. The residue-covered filter paper was dried in an oven at 105°C for 3 h or until it reached a consistent weight. It was weighed after cooling in a desiccator. The weight decrease was due to the crude fiber content which was calculated by using Equation (3).


(3) $$\begin{array}{r} Crude\;fiber\;\left(\%\right)=\frac{M_{2}-M_{1}}{Mass\;of\;sample}\times 100 \end{array}$$

where M_1_ = mass of filter paper and M_2_ = mass of residue and filter paper.

#### Total soluble solids

The amount of total soluble solids in the sample was assessed through Abbe's refractometer by putting a drop of the extracted sample of Indian mustard on the prism and taking the reading (AOAC, 1995).

#### Percentage of protein

Protein percent in Indian mustard was evaluated by multiplication of nitrogen content (measured by digesting samples with H_2_SO_4_ and total nitrogen was calculated by micro Kjeldahl's technique presented by Jackson, 1967) with the factor of 6.25.

### Yield analysis

Seed and stover yield were measured from the net plot area leaving the border rows and were later converted to Kg ha^−1^. To measure the dry weight of different components of the plant, the samples were air-dried before drying in an oven at 65°C for 48 h. A mechanical grinder was used to grind the plant samples to a fine powder. The grounded samples of stover and seed weighing 1.0 and 0.5 g, respectively, were subjected to digestion using a mixture of di-acid, i.e., HNO_3_ and HClO_4_ acid in a 3:1 ratio on a hot plate (Kumar and Dhaliwal, 2021). The B, S, and N contents in digested extracts of the plant were measured using an atomic absorption spectrophotometer (Model AAS 240 FS, Company Varian, Germany), whereas B, S, and N uptakes by seed and stover of Indian mustard (g ha^−1^) were calculated by using Equation (4).


(4) $$\begin{array}{l} \text{ Nutrient uptake }\left(\text{g }{\text{ha}}^{\text{-1}}\right)\\ =\frac{\text{concentration }\left(\text{mg }{\text{kg}}^{\text{-1}}\right)\times \text{Yield}(\text{Kg }{\text{ha}}^{\text{-1}})}{1000} \end{array}$$

### Nutrient use efficiency indices

The determination of nutrient use efficiency indices for B, S, and N including apparent recovery efficiency (ARE) and mobilization efficiency index (MEI) was done as per Equations (5) and (6), respectively (Dhaliwal et al., 2021c).


(5) $$\begin{array}{rcl} ARE & = & \frac{\;NUt-\;NUc}{Nutrient\;applied\;\left(kg/ha\right)}\times 100 \end{array}$$

(6) $$\begin{array}{rcl} MEI & = & \frac{Concentration\;of\;nutrient\;in\;seed\;\left(\text{mg kg}-1\right)}{Concentration\;of\;nutrient\;in\;stover\left(\text{mg kg}-1\right)}, \end{array}$$

where NU_t_ and NU_c_ in Equation (5) denote the total B, S, and N uptake (g ha^−1^) by Indian mustard in B and N fertilized plots and in control, respectively.

### Economics

The cost of fertilizer in United States Dollars (USD) per hectare was calculated independently for each treatment in the experiment, taking into account current fertilizer costs in USD at the time of application. During the study period, the gross return (value of additional output) was computed using the MSP (minimum support price) of Indian mustard set by the Indian government (Eqsuations 7 and 8).


(7) $$\begin{array}{r} Grossreturn=Yield\times Priceofproduce \end{array}$$

(8) $$\begin{array}{l} \text{Net Return }\left(\text{USD }{\text{ha}}^{\text{-1}}\right)\text{=Gross return }\left(\text{USD }{\text{ha}}^{\text{-1}}\right)\\ \text{ - Cultivation cost }\left(\text{USD }{\text{ha}}^{\text{-1}}\right)\\ \text{ B}:\text{C ratio= }\frac{\text{Gross return }\left(\text{USD }{\text{ha}}^{\text{-1}}\right)}{\text{Cultivation cost }\left(\text{USD }{\text{ha}}^{\text{-1}}\right)} \end{array}$$

### Statistical analysis

Data were analyzed statistically using the statistical package SPSS version 16.0 (SPSS Inc., Chicago, USA) packages (SPSS Inc., 2007). All parameters were studied using a one-way analysis of variance (ANOVA) for comparing means and differences among the treatments by using the Duncan Multiple Range Test (DMRT) at a 0.05 probability level.

## Results

### Foliar application of B and N affecting some quality and growth parameters of Indian mustard

Results on the quality parameters of mustard seed powder are presented in Table 1. It was found that the application of B and N significantly improved the quality parameters in Indian mustard where the amount of moisture in mustard seeds for all the treatments ranged from 3.92 to 5.0%. The highest moisture content (5.0%) was observed in treatment T_9_ involving borax (1.0%) + urea (1.0%) at the flowering and capsule formation stage followed by treatment T_8_ (4.80%) involving borax (0.5%) + urea (1.0%) at the flowering + capsule formation stage. However, the total percentage of ash varied from 3.74 to 4.88% where the ash content was highest in treatment T_9_ involving borax (1.0%) + urea (1.0%) at the flowering and capsule formation stage, whereas the lowest ash content was found in treatment T_1_, i.e., control. Additionally, the crude fiber content varied from 1.78 to 3.77%. The increment in the content of crude fiber was observed with the B and N application where treatment T_9_ involving borax (1.0%) + urea (1.0%) at the flowering and capsule formation stage had the maximum (3.77%) and treatment T_1_, i.e., control had the minimum amount of crude fiber (1.78%), respectively. Moreover, the treatments T_9_ (3.77%) and T_8_ (3.58%) were statistically at par with each other. Also, there was a significant increase in the TSS value with the application of B and N over the control. The highest value (24.9%) was observed in treatment T_9_ whereas the lowest was observed in treatment T_1_ (18.4%). Furthermore, the results in Table 1 showed that the protein content in mustard seed was highest for Treatment T_9_ (27.53%) and lowest for treatment T_1_, i.e., control (19.13%). Also, the results of treatment T_9_ were not statistically different from treatment T_8_ (26.95).

**Table 1:** Impact of foliar application of B and N on the chemical composition of Indian mustard.

**Treatments**	**Moisture (%)**	**Ash (%)**	**Crude fiber (%)**	**Total soluble solids (%)**	**Protein (%)**
T_1_	3.92^e^	3.74^h^	1.78^g^	18.4^f^	19.13^e^
T_2_	4.23^d^	3.96^g^	2.24^f^	19.5^ef^	21.23^d^
T_3_	4.44^c^	4.10^f^	2.55^e^	20.6^de^	21.47^d^
T_4_	4.50^c^	4.40^d^	3.08^bc^	22.7^bc^	25.67^ab^
T_5_	4.70^b^	4.53^c^	3.25^b^	22.9^bc^	26.60^a^
T_6_	4.41^c^	4.11^e^	2.72^de^	21.7^cd^	22.87^cd^
T_7_	4.52^c^	4.22^e^	2.98^c^	22.0^cd^	24.26^bc^
T_8_	4.80^b^	4.71^b^	3.58^a^	23.8^ab^	26.95^a^
T_9_	5.00^a^	4.88^a^	3.77^a^	24.9^a^	27.53^a^
LSD (0.05)	0.16	0.11	0.19	1.40	1.95

The column mean with a similar or dissimilar letter(s) was evaluated with the least significant difference (LSD) multiple range tests using a probability level of a p-value ≤ 0.05. T_1_-Control (recommended NPK only), T_2_- borax (0.5%) at flowering, T_3_-borax (1.0%) at flowering,T_4_- borax (0.5%) + urea (1.0%) at flowering,T_5_-borax (1.0%) + urea (1.0%) at flowering, T_6_-borax (0.5%) at flowering + capsule formation, T_7_-borax (1.0%) at flowering + capsule formation, T_8_-borax (0.5%) + urea (1.0%) at flowering + capsule formation, T_9_-borax (1.0%) + urea (1.0%) at flowering + capsule formation.

**Figure 1:**
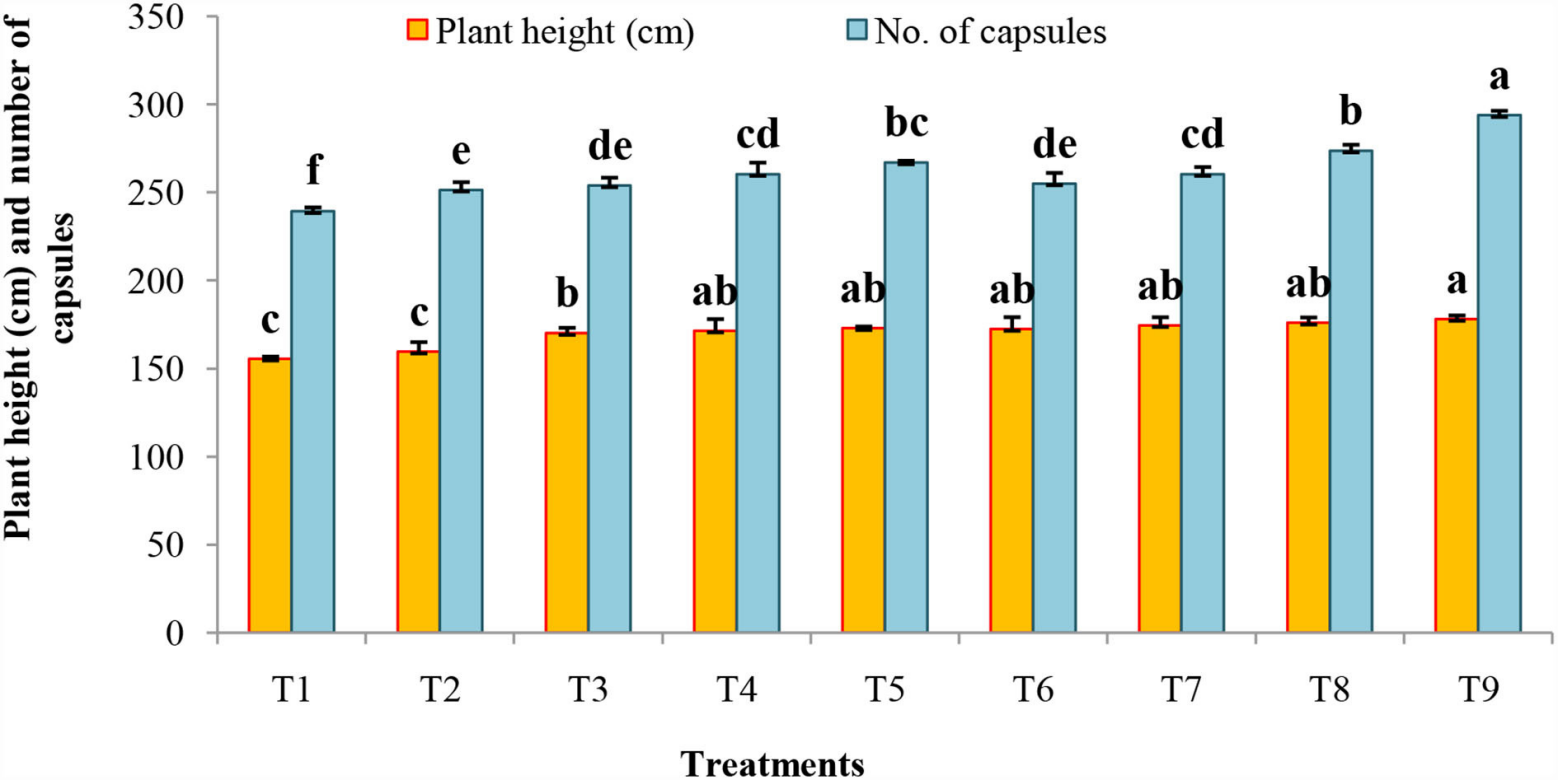
Impact of foliar application of B and N on biometrical parameters of Indian mustard during 2020–2021 and 2021–2022.

The B and N fertilization contributed to a great extent in influencing the growth parameters such as plant height and the number of capsules in Indian mustard at various stages of the crop growth (Figure 1).

It was observed that the plant height increased with the advancement in crop growth up to treatment T_9_ (178.04 cm) involving borax (1.0%) + urea (1.0%) at the flowering and capsule formation stage, which was statistically at par with treatment T_8_ (175.94 cm). Significantly, lower plant height in treatment T_1_ (155.54 cm) as compared to the rest of the treatments indicated the importance of B in plant growth. Similarly, treatment T_9_ was significantly superior to the rest of the treatments having the maximum number of capsules (293.77), while minimum capsules, i.e., 239.22 were counted in treatment T_1_ (control).

### Foliar application of B and N affecting seed as well as stover yield of Indian mustard

The data on yield demonstrated that the application of B and N posed a significant impact on the yield of seed as well as stover in Indian mustard (Table 2).

**Table 2:** Impact of foliar application of B and N on seed and stover yield of Indian mustard during 2020–2021 and 2021–2022.

**Treatments**	**Seed yield (kg ha** ^**−1**^ **)**			**Stover yield (kg ha** ^**−1**^ **)**			**Harvest index (%)**
	**Year I**	**Year II**	**Mean**	**Year I**	**Year II**	**Mean**	
T_1_	956^f^	1100^d^	1028^e^	3477^g^	4383^f^	3930^g^	20.73^a^
T_2_	1172^e^	1213^c^	1193^d^	4544^f^	5287^e^	4916^f^	19.53^b^
T_3_	1184^de^	1242^c^	1213^cd^	4766^ef^	5442^de^	5104^ef^	19.20^b^
T_4_	1272^abcd^	1295^bc^	1284^bc^	5461^cd^	6138^bc^	5799^cd^	18.13^c^
T_5_	1293^abc^	1333^ab^	1313^ab^	5707^bc^	6433^b^	6070^bc^	17.78^de^
T_6_	1208^cde^	1247^c^	1227^cd^	5126^de^	5728^cde^	5427^de^	18.44^c^
T_7_	1253^bcde^	1262^bc^	1258^bcd^	5313^cd^	5922^cd^	5618^cd^	18.29^cd^
T_8_	1307^ab^	1350^ab^	1329^ab^	6026^ab^	6600^ab^	6313^ab^	17.39^ef^
T_9_	1348^a^	1403^a^	1376^a^	6319^a^	6930^a^	6625^a^	17.19^f^
LSD (0.05)	88.2	99.3	80.9	405.3	489.2	478.4	0.57

The column mean with a similar or dissimilar letter(s) was evaluated with the least significant difference (LSD) multiple range tests using a probability level of a p-value ≤ 0.05. T_1_-Control (recommended NPK only), T_2_-Borax (0.5%) at flowering, T_3_-borax (1.0%) at flowering, T_4_-borax (0.5%) + urea (1.0%) at flowering, T_5_-borax (1.0%) + urea (1.0%) at flowering, T_6_-borax (0.5%) at flowering + capsule formation, T_7_-borax (1.0%) at flowering + capsule formation, T_8_-borax (0.5%) + urea (1.0%) at flowering + capsule formation, and T_9_-borax (1.0%) + urea (1.0%) at flowering + capsule formation.

The minimum value of seed and stover yield was observed in treatment T_1_, i.e., control with mean values of 1,028 and 3,930 kg ha^−1^, respectively. Sole application of B in treatments T_2_, T_3_, T_6_, and T_7_ does not show a remarkable increase in seed and stover yields of Indian mustard. However, the addition of borax (0.5%) along with urea (1.0%) at the flowering and capsule formation stage (T_8_) significantly improved the seed and stover yields to 1,329 and 6,313 kg ha^−1^ with an increase of 22.6 and 37.7% over control, respectively. Whereas, the maximum values of 1,376 and 6,625 kg ha^−1^ for seed and stover yield were observed in treatment T_9_ involving borax (1.0%) + urea (1.0%) at the flowering and capsule formation stage with the increased dose of borax, which was statistically at par with treatment T_8_. The highest percentage increase in seed, as well as stover yield in treatment T9, was observed to be 25.2 and 40.7% over control, respectively. Data pertinent to the harvest index as influenced by different treatments of B and N are presented in Table 2, where the values varied from 17.19 to 20.73.

### Foliar application of B and N affecting B, S, and N concentrations and their uptake in Indian mustard

The mean of 2 years (2020–2021 and 2021–2022) data on the concentration of B, S, and N in seed and stover of Indian mustard is presented in Table 3.

**Table 3:** Impact of foliar application of B and N on the concentrations of B, S, and N in seed and stover of Indian mustard during 2020–2021 and 2021–2022.

**Treatments**	**Seed concentration**			**Stover concentration**		
	**B (mg kg^**−1**^)**	**S (mg kg^**−1**^)**	***N (%)***	**B (mg kg^**−1**^)**	**S (mg kg^**−1**^)**	***N (%)***
T_1_	16.17^g^	5.06^g^	1.61^g^	14.68^g^	5.00^g^	0.19^f^
T_2_	18.46^f^	9.23^f^	1.71^fg^	16.94^fg^	8.79^f^	0.21^ef^
T_3_	19.12^f^	10.67^ef^	1.79^ef^	17.57^ef^	9.48^ef^	0.22^ef^
T_4_	22.39^cd^	13.92^c^	2.04^bcd^	20.63^cd^	12.59^cd^	0.27^cd^
T_5_	23.12^c^	14.36^bc^	2.13^bc^	22.35^bc^	13.64^bc^	0.28^bc^
T_6_	19.71^ef^	11.75^de^	1.92^de^	18.27^def^	10.66^def^	0.23^e^
T_7_	21.00^de^	12.74^cd^	2.00^cd^	19.62^de^	11.69^de^	0.24^de^
T_8_	25.39^b^	16.16^b^	2.19^ab^	23.78^ab^	14.66^b^	0.31^ab^
T_9_	27.71^a^	17.69^a^	2.35^a^	25.92^a^	17.31^a^	0.33^a^
LSD (0.05)	1.79	1.84	0.17	2.52	2.45	0.03

The column mean with a similar or dissimilar letter(s) was evaluated with the least significant difference (LSD) multiple range tests using a probability level of a p-value ≤ 0.05. T_1_-Control (recommended NPK only), T_2_-borax (0.5%) at flowering, T_3_-borax (1.0%) at flowering, T_4_-borax (0.5%) + urea (1.0%) at flowering, T_5_-borax (1.0%) + urea (1.0%) at flowering, T_6_-borax (0.5%) at flowering + capsule formation, T_7_-borax (1.0%) at flowering + capsule formation, T_8_-borax (0.5%) + urea (1.0%) at flowering + capsule formation, and T_9_-borax (1.0%) + urea (1.0%) at flowering + capsule formation.

All combinations of B and N resulted in the increased concentration of B, S, and N in seed and stover in comparison to the control. Maximum concentrations of B, S, and N in both seed, as well as stover, were recorded with two foliar sprays of B and N at the flowering and capsule formation stage in treatment T_9_ possessing the highest concentrations of 27.71 mg Kg^−1^, 17.69 mg Kg^−1^, and 2.35% for seed and 25.92 mg Kg^−1^, 17.31 mg Kg^−1^, and 0.33% for stover, respectively. The results of treatment T_9_ for B and N stover concentrations were statistically at par with treatment T_8_, whereas the minimum concentration was observed in treatment T_1_, i.e., control. Additionally, B, S, and N concentrations in seeds of Indian mustard were more than those found in stover indicating the enhanced translocation of nutrients into the seed from stover. Data on the micronutrient uptake in Indian mustard affected by foliar use of B and N are given in Table 4.

**Table 4:** Impact of foliar application of B and N on the uptake of B, S, and N in seed and stover of Indian mustard during 2020–2021 and 2021–2022.

**Treatments**	**Uptake in seed**			**Uptake in stover**		
	**B (g ha^**−1**^)**	**S (g ha^**−1**^)**	***N (Kg ha^**−1**^)***	**B (g ha^**−1**^)**	**S (g ha^**−1**^)**	***N (Kg ha^**−1**^)***
T_1_	16.71^g^	5.29^g^	15.92^h^	58.18^g^	19.81^g^	7.54^e^
T_2_	22.04^f^	11.03^f^	20.20^g^	83.59^f^	43.48^f^	10.34^d^
T_3_	23.23^f^	12.97^ef^	21.55^fg^	89.94^f^	48.77^ef^	11.94^cd^
T_4_	28.77^cd^	17.89^c^	26.12^cd^	120.33^cd^	73.65^bcd^	15.73^b^
T_5_	30.39^c^	18.89^bc^	27.79^bc^	136.49^bc^	83.39^bc^	17.29^b^
T_6_	24.22^ef^	14.44^de^	23.35^ef^	99.49^ef^	58.27^de^	12.39^cd^
T_7_	26.41^de^	16.03^cd^	25.13^de^	110.69^de^	66.16^cde^	13.08^c^
T_8_	33.79^b^	21.52^ab^	28.91^b^	150.87^b^	93.29^b^	20.09^a^
T_9_	38.18^a^	24.40^a^	32.05^a^	172.55^a^	115.44^a^	21.99^a^
LSD (0.05)	2.88	2.94	2.18	20.70	19.69	2.06

The column mean with a similar or dissimilar letter(s) was evaluated with the least significant difference (LSD) multiple range tests using a probability level of a p-value ≤ 0.05. T_1_-Control (recommended NPK only), T_2_-borax (0.5%) at flowering, T_3_-borax (1.0%) at flowering, T_4_-borax (0.5%) + urea (1.0%) at flowering, T_5_-borax (1.0%) + urea (1.0%) at flowering, T_6_-borax (0.5%) at flowering + capsule formation, T_7_-borax (1.0%) at flowering + capsule formation, T_8_-borax (0.5%) + urea (1.0%) at flowering + capsule formation, and T_9_-borax (1.0%) + urea (1.0%) at flowering + capsule formation.

The results demonstrated that the uptake of nutrients was significantly influenced by the foliar application of B and N over control. In seed and stover, B uptake varied from 16.71 to 38.18 g ha^−1^ and 58.18 to 172.55 g ha^−1^, respectively. Similarly, S and N uptakes were found to range between 5.29 and 24.40 g ha^−1^ and between 15.92 and 32.05 Kg ha^−1^, for seeds whereas, the values were found to vary between 19.81 and 115.44 g ha^−1^ and between 7.54 and 21.99 Kg ha^−1^ for stovers, respectively. Treatment T_9_ involving foliar application of borax (1.0%) + urea (1.0%) at the flowering and capsule formation stage exhibited maximum nutrient uptake followed by treatment T_8_ with B, S, and N uptake values of 33.79 g ha^−1^, 21.52 g ha^−1^, and 28.91 Kg ha^−1^ in seeds and 150.87 g ha^−1^, 93.29 g ha^−1^, and 20.09 Kg ha^−1^ in stovers, respectively. The results of treatments T_8_ and T_9_ were statistically at par with each other for S in the seeds and for N in the stovers of Indian mustard.

### Foliar application of B and N affecting efficiency indices and economic outcomes of Indian mustard

Different efficiency indices in Table 5 shows that the ME-B, ME-S, and ME-N were highest in T_9_ (1.10, 1.13, and 8.58%) and lowest (1.02, 1.01, and 6.88%) in T_1_. Moreover, ME was higher in the presence of borax (1.0%) plus urea (1.0%) at the flowering and capsule formation stage, whereas ME values were less in treatments involving sole application of B and N. Again, ARE-B, ARE-S, and ARE-N were highest in treatment T_9_ (0.61, 1.1, and 158.78%), whereas the least value for ARE were seen in treatment T_1_, i.e., control.

**Table 5:** Impact of foliar application of B and N on nutrient use efficiencies by Indian mustard during 2020–2021 and 2021–2022.

**Treatments**	**Mobilization efficiency index (%)**			**Apparent Recovery efficiency (%)**		
	**B**	**S**	***N***	**B**	**S**	***N***
T_1_	1.02	1.01	6.88	–	–	–
T_2_	1.05	1.05	7.09	0.33	0.4	152.70
T_3_	1.06	1.06	8.05	0.36	0.5	155.56
T_4_	1.07	1.09	8.13	0.44	0.7	164.53
T_5_	1.08	1.10	7.49	0.48	0.8	177.33
T_6_	1.06	1.08	7.55	0.36	0.6	232.71
T_7_	1.07	1.09	7.70	0.38	0.7	146.22
T_8_	1.09	1.11	8.38	0.40	0.9	161.82
T_9_	1.10	1.13	8.58	0.61	1.1	158.78

T_1_-Control (recommended NPK only), T_2_-borax (0.5%) at flowering, T_3_-borax (1.0%) at flowering, T_4_-borax (0.5%) + urea (1.0%) at flowering, T_5_-borax (1.0%) + urea (1.0%) at flowering, T_6_-borax (0.5%) at flowering + ccapsule formation, T_7_-borax (1.0%) at flowering + capsule formation, T_8_-borax (0.5%) + urea (1.0%) at flowering + capsule formation, and T_9_-borax (1.0%) + urea (1.0%) at flowering + capsule formation.

**Figure 2:**
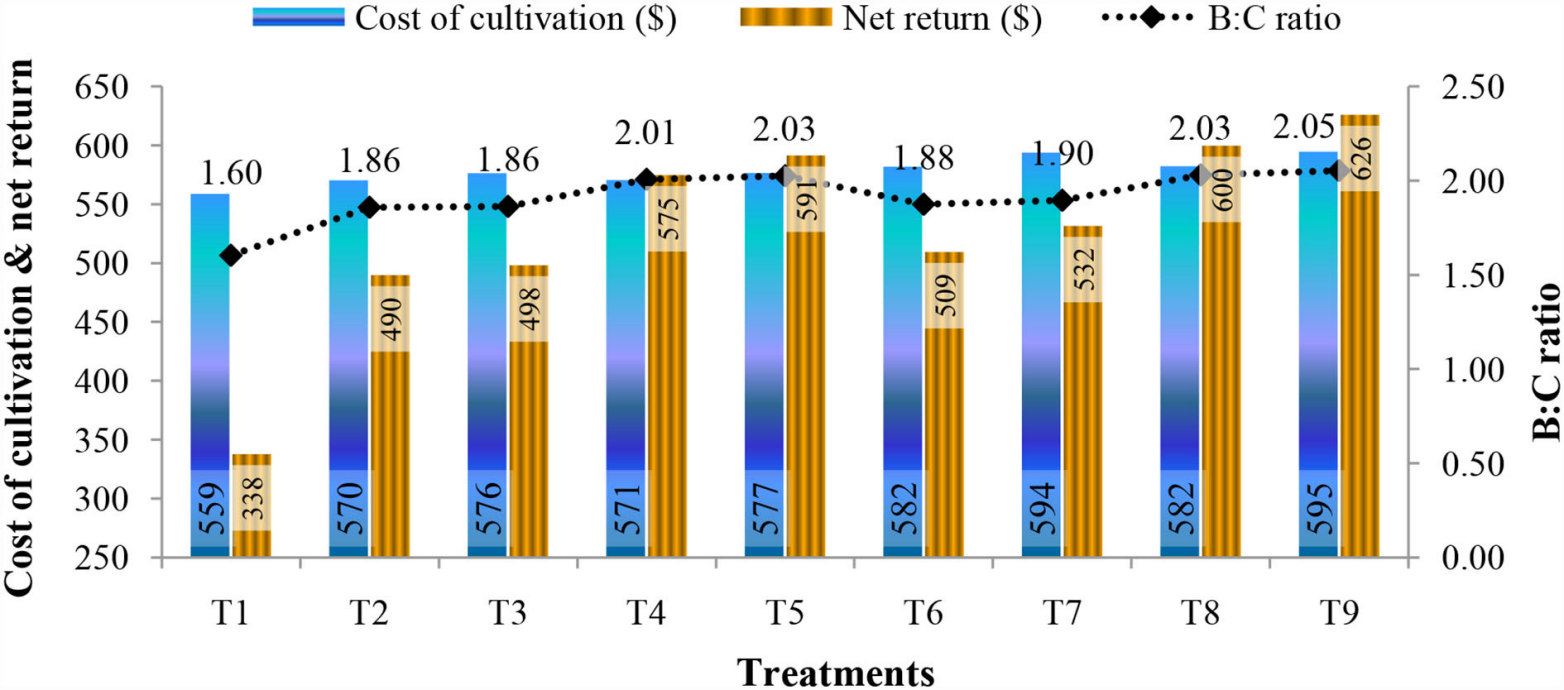
Foliar application of B and N affecting cultivation cost, net return, and B:C (benefit:cost ratio) of Indian mustard during 2020–2021 and 2021-2022.

The effects of B and N foliar application at the flowering and capsule formation stage on the economics of Indian mustard cultivation is given in Figure 2. The cultivation cost, net return, and benefit:cost ratio were affected positively by B and N treatments. Data displayed that the highest cultivation cost was found in treatment T_9_ ($595) followed by treatment T_7_ ($594), whereas the lowest cultivation cost was found in treatment T_1_ ($559). The highest value of net return was calculated in treatment T_9_ ($626) followed by T_8_ ($600). Also, the benefit:cost ratio was highest in T_9_ (2.05) followed by T_8_ and T_5_ (2.03) and lowest in control (1.60).

## Discussion

### Foliar application of B and N affecting some quality and growth parameters of Indian mustard

In the present study, the results on the moisture content showed that the highest value was observed in the treatment involving combined foliar application of B and N at the flowering and capsule formation stage. These results corroborated well with the studies conducted by AlMahmud et al. (2012) and Sarker et al. (2015). These variations in moisture contents under the treatments involving borax and urea were mainly due to the different levels of sun drying after harvesting. However, the ash content in the present study was in agreement with values reported by Abul-Fadl et al. (2011) and Sarker et al. (2015). The percentage increase in crude fiber with the foliar use of B and N over the control might be due to the role of B in protein metabolism, which affects fiber properties (Kumar and Dhaliwal, 2021). One of the studies presented the values of fiber content to be 5.87 and 6.34% for yellow and brown mustard seed flour, respectively (Abul-Fadl et al., 2011). The increase in TSS values with the foliar application of N could be due to the increased photosynthesis and carbohydrate production in the crop (Marschner, 1995). However, the enhanced level of TSS through B treatment was attributed to the role of B in carbohydrate metabolism, which was focused on the synthesis of the cell wall material and the transport of sugars (Marschner, 1995). The higher protein content under different treatments as compared to control was due to more availability of N, which increased proteinase substances in the seed under high N supply where a large proportion of photosynthates may have diverted to protein formation. Moreover, increased seed, as well as stover yield, further resulted in higher protein content. On the contrary, B holds a major role in protein metabolism through several enzyme systems and the translocation of proteins to seeds, which improves the total protein content in Indian mustard (Roy et al., 2013).

The improved value of plant height in treatment T_9_ might be attributed to the involvement of B in hormone synthesis and translocation, carbohydrate metabolism, and the synthesis of DNA in plants (Verma et al., 2020). Identical results for plant height were reported by Shah et al. (2016) and Handiganoor et al. (2017). Whereas, the increased number of capsules with the foliar application of B and N observed in the present study might be due to the involvement of B in reproductive growth as B improves the fertility in plants. In addition to this, improvement in the growth and yield attributes of Indian mustard due to N application appeared quite logical (Dhaliwal et al., 2022). It is well-known that N being the constituent of amino acids, proteins, chlorophyll, and protoplast would directly influence the growth and yield attributing characteristics through better utilization of photosynthates. Also, the increase in N supply increases the amount of protein formed and therefore the amount of protoplasm. This increase in turn results in greater cell size, more cell division, and larger leaf area. It ultimately provides a larger frame, resulting in improved plant height and number of capsules. Singh and Kumar (2014) also reported an increase in growth and yield attributes of rapeseed-mustard due to N application. Thus, the synergistic effect of borax and urea further improved the yield attributes in Indian mustard.

### Foliar application of B and N affecting seed as well as stover yield of Indian mustard

The reason for the lowest seed and stover yield in B and N deprived plots, that is, treatment T_1_ might be the higher pollen infertility and lower seed filling as they play an extremely crucial role in both the processes (Hussain et al., 2012). The highest seed yield with an increased rate of B and N fertilization might be ascribed to an increased number of fertile pods resulting in higher seed production and yield (Randhawa et al., 2021). Boron's involvement in maintaining the structural integrity of plasma membrane (Handiganoor et al., 2017), metabolism of carbohydrates, and synthesis of DNA (Davidson, 1987) probably resulted in the additional growth in crops. Moreover, the seed and stover yield improvement in Indian mustard was linked to the integral role of B in elongation and division of cells along with biomass accumulation (Jaiswal et al., 2015). Additionally, B might be involved in the synthesis of protein, chloroplast pigments, and electron transfer that led to the increased photosynthetic activity of Indian mustard, thus accounting for higher yield (Srinivasan et al., 2019). The positive effect of B application on mustard yield has been reported in Bangladesh by Haque et al. (2000) and Islam (2005). Additionally, the increase in yield of mustard due to N application might be because of the fact that N plays an important role in the synthesis of chlorophyll and amino acids, which are the important building blocks of protein synthesis. Nitrogen influenced the seed yield through a source-sink relationship and in addition to higher production of photosynthates, it resulted in increased translocation to reproductive parts. Nitrogen being the most important plant nutrient needed for the growth and development of plants was known to increase the yield of *Brassica* species (Singh et al., 2002). Thus, the combined foliar application of B and N resulted in significant improvement in seed as well as stover yield in Indian mustard.

### Foliar application of B and N affecting B, S, and N concentrations and their uptake in Indian mustard

The increase in B, S, and N concentrations with the combined foliar application of B and N might be associated with the enhanced bioavailability of these nutrients. Also, N helped in synthesizing the nutrient regulator protein, thereby increasing the translocation of B and S in the crop (Jankowski et al., 2019). Furthermore, the increased absorption, as well as assimilation of B and N, resulted in balanced nutritional value in the crop for higher growth and thereby higher nutrient content. Fertilization with borax (1.0%) along with urea (1.0%) at the flowering and capsule formation stage (T_9_) led to a significant increase in nutrient absorption and transfer by mustard, and that might be due to the enhanced root growth, resulting in increased absorption by root tips and its transfer to the phloem (Lakshmanan et al., 2005). One of the previous studies reported the increased micronutrient concentrations in Indian mustard on an increase in the application rate of B (Kumararajaa et al., 2015).

Additionally, the improved B, S, and N uptake through the foliar application of B and N could be related to the joint impact of yield as well as concentration. Moreover, the exogenous supply of B and N through foliar application had a noticeable influence on their availability in Indian mustard. The highest nutrient uptake at borax (1.0%) along with urea (1.0%) was due to the increased yield, nutrient concentration, nutrient mobility, and absorption. Thus, the extensive root system development with a higher rate of B and N application in adequate amounts might have assisted in the efficient absorption and distribution of nutrients in different parts of the crop, thus increasing nutrient uptake. The increased availability of these nutrients in the root zone coupled with increased metabolic activity at cellular levels might have synthesized more nutrients and increased their accumulation in various plant parts (Shrama et al., 2017). These results are in agreement with studies conducted by Jat et al. (2012) and Kansotia et al. (2013).

### Efficiency indices and economic analysis of Indian mustard

The results of ME demonstrated that the application of borax and urea at an increased rate (1.0%) resulted in more nutrient translocation toward the seed as compared to stover. The lower value of ME suggested the enhanced nutrient translocation in stovers as compared to the seeds. Again, ARE-B, ARE-S, and ARE-N were highest in treatment T_9_, whereas the least value for ARE was seen in treatment T_1_, i.e., control. This showed that maximum nutrient absorption by the plant was achieved with foliar application of borax and urea (1.0%) at the flowering and capsule formation stage. Similar results of the increase in efficiency indices with Zn, Fe, and N application were reported in Indian mustard (Dhaliwal et al., 2021a).

In terms of economics, the results displayed that the highest cultivation cost was found in treatment T9, whereas the lowest cultivation cost was found in treatment T_1_. Similarly, the highest value of net return was calculated in treatment T_9_ followed by T8. Also, the benefit:cost ratio was highest in T_9_ followed by T_8_, and lowest in control. Thus, the foliar use of B and N through borax and urea enhanced the economic outcomes of Indian mustard. These outcomes were in agreement with the earlier results in which Zn, Fe, and N treatments resulted in an enhanced B:C ratio of Indian mustard (Dhaliwal et al., 2021a). Additionally, the combined application of borax (1.0%) and urea (1.0%) at the flowering and capsule formation stage exhibited greater net return and B:C ratio, which proves its effectiveness over other treatments and control.

## Conclusion

Indian mustard is an important oilseed crop that can retain and enhance the productivity and nutrient quality of the crop through biofortification for higher food quality. The current study aimed to determine the most efficient and economical B and N treatments to enhance the oil quality, yield, and concentration, as well as uptake of nutrients in Indian mustard. Boron, as well as N foliar application, improved the proximate composition, including crude fiber and TSS levels in oil seeds of the crop, which are considered to be essential for enhancing the nutritional level in Indian mustard. Also, the results demonstrated the increased growth parameters as well as yield attributes, which further helped in increasing the oil seed quality. This is the first study that evaluated the effect of the sole as well as combined foliar application of B and N on the nutritional quality of Indian mustard. Treatment T_9_ involving the combined use of borax (1.0%) and urea (1.0%) at the flowering and capsule formation stage proved highly beneficial, which also possessed economically superior outcomes based on higher net return and B:C values over other treatments. Thus, this study suggests the need for adequate levels of B and N to enhance the nutritional quality of Indian mustard, which could be used to improve the food quality of other oil seed crops, which in turn would improve the health of consumers through potential reduction of B deficiency and increased intake of S and other useful nutrients.

## Data availability statement

The raw data supporting the conclusions of this article will be made available by the authors, without undue reservation.

## Author contributions

SSD, MK, VV, PSS, VS, and AKS: methodology. SSD, VS, VV, and MK: software. SSD, PSS, and AH: validation. SSD, VS, VV, MK, and PSS: formal analysis. SSD and AH: investigation. SSD and VS: resources. MK: data curation. SSD, PSS, and AH: writing-original draft preparation. SSD, VS, and AKS: writing-review and editing. AG and AH: visualization. SSD, PSS, and VS: supervision. SSD, VS, AKS, and VV: project administration. SSD, AG, AMA, and AH: funding acquisition.

## Funding

This research was funded by the Department of Soil Science, Punjab Agricultural University, Ludhiana, India Ludhiana, India and ICAR-Indian Institute of Soil Science, Bhopal, 462038, Madhya Pradesh, India. This research was also partially funded by the Princess Nourah bint Abdulrahman University Researchers Support Project number (PNURSP2022R65) of Princess Nourah bint Abdulrahman University, Riyadh, Saudi Arabia.

## Conflict of interest

The authors declare that the research was conducted in the absence of any commercial or financial relationships that could be construed as a potential conflict of interest.

## Publisher's note

All claims expressed in this article are solely those of the authors and do not necessarily represent those of their affiliated organizations, or those of the publisher, the editors and the reviewers. Any product that may be evaluated in this article, or claim that may be made by its manufacturer, is not guaranteed or endorsed by the publisher.
